# Pan-European Training Program for Robotic Pancreatoduodenectomy (LEARNBOT): Prospective Multicenter Study on Feasibility and Outcome

**DOI:** 10.1097/SLA.0000000000007170

**Published:** 2026-10-07

**Authors:** Leia R. Jones, Maurice J.W. Zwart, Felix Nickel, Thilo Hackert, Freek Daams, Bas Groot Koerkamp, Quintus Molenaar, Ugo Boggi, Olivier Saint-Marc, Philip Leenart, Rawin Amiri, Mahsoem Ali, Sebastiaan Festen, Olivier Busch, Paul S. Krohn, Ernesto Sparrelid, Bergthor Björnsson, Christian Toso, Edoardo Rosso, Benjamin Strücker, Peter Strandberg-Holka, Maxime Dewulf, Maximilian Schmeding, Emre Bozkurt, Giovanni Ferrari, Steve White, Emanuel Vigia, Zahir Soonawalla, Martijn Stommel, Tim Pencavel, Martin Lovecek, Declan Dunne, Giovanni Marchegiani, Filip Gryspeerdt, Melissa E. Hogg, Mohammad Abu Hilal, Marc G. Besselink

**Affiliations:** 1 Department of Surgery, Amsterdam UMC, location University of Amsterdam, The Netherlands; 2 Cancer Center Amsterdam, The Netherlands; 3 Department of General Surgery, Instituto Ospedaliero Fondazione Poliambulanza, Brescia, Italy; 4 Department of Surgery, Universitätsklinikum Hamburg-Eppendorf, Hamburg, Germany; 5 Department of Surgery, Amsterdam UMC, location Vrije Universiteit, The Netherlands; 6 Department of Surgery, Erasmus Medical Center, Rotterdam, The Netherlands; 7 Department of Surgery, University Medical Center Utrecht, Utrecht, The Netherlands; 8 Division of General and Transplant Surgery, University of Pisa, Pisa, Italy; 9 Department of Digestive and Endocrine Surgery, University Hospital of Orléans, Orléans, France; 10 Department of Surgery, OLVG, Amsterdam, The Netherlands; 11 Department of Surgery, Rigshospitalet, Copenhagen, Denmark; 12 Department of Clinical Science, Intervention and Technology, Department Division of Surgery and Oncology, Karolinska Institutet, Karolinska University Hospital, Stockholm, Sweden; 13 Department of Surgery and Department of Biomedical and Clinical Sciences, Linköping University, Linköping, Sweden; 14 Department of Surgery, Geneva University Hospitals, Geneva, Switzerland; 15 Department of Surgery, Centre Hospitalier de Luxembourg, Luxembourg, Luxembourg; 16 Department of Surgery, University Hospital Münster, Münster, Germany; 17 Department of Surgery, Skane University Hospital, Lund, Sweden; 18 Department of Surgery, Maastricht University Medical Center, Maastricht, The Netherlands; 19 Department of Surgery, Klinikum Dortmund, University of Witten/Herdecke, Dortmund, Germany; 20 Department of General Surgery, Koc University School of Medicine, Istanbul, Turkey; 21 Department of Surgery, Niguarda Hospital, Milan, Italy; 22 Department of Surgery, The Newcastle upon Tyne Hospitals NHS Foundation Trust, Newcastle, UK; 23 Department of Surgery, Curry Cabral Hospital, Lisbon, Portugal; 24 Department of Surgery, Oxford University Hospitals NHS Foundation Trust, Oxford, UK; 25 Department of Surgery, Radboud University Medical Center, Nijmegen, The Netherlands; 26 Department of Surgery, Royal Surrey NHS Foundation Trust, Guildford, UK; 27 Department of Surgery, Olomouc University Hospital, Olomouc, Czech Republic; 28 Department of Surgery, Royal Liverpool University Hospital NHS Foundation Trust, Liverpool, UK; 29 Department of Surgery, Oncology and Gastroenterology, University of Padova, Padova, Italy; 30 Department of Surgery, Ghent University Hospital, Ghent, Belgium; 31 Department of Surgery, Northshore University Health System, Chicago, IL; 32 Department of Surgery, School of Medicine, The University of Jordan, Amman, Jordan; 33 Department of Surgery, University Hospital Southampton NHS Foundation Trust, Southampton, UK

**Keywords:** RPD, training program, learning curve, robot surgery, pancreatoduodenectomy, pancreas

## Abstract

**Objective:**

To assess the feasibility of and clinical outcomes after a pan-European training program for robotic pancreatoduodenectomy (RPD).

**Summary Background Data:**

The implementation of RPD into clinical practice comes with considerable challenges, leading to concerns about patient safety, oncological outcomes, and costs. To address this, a structured training program was implemented in which 2 surgeons at each of the 20 sites were trained and maintained as a consistent operative team.

**Study Design:**

A structured training program for RPD was implemented, and outcomes were prospectively collected in 20 European high-volume centers, all without previous RPD experience, from 12 countries. The program included a video library, biotissue simulation (pancreaticojejunostomy and hepaticojejunostomy), off-site RPD case observations, and on-site proctoring. Surgeons’ simulation anastomoses were assessed using Objective Structured Assessment of Technical Skills (OSATS) and technical errors. The primary endpoint was intra- and postoperative patient outcome. A key secondary analysis assessed safety using OSATS scores and error counts during the biotissue training..

**Results:**

Overall, 486 RPD procedures were performed. Median intraoperative blood loss was 200 mL (IQR: 100–400), operative time was 475 minutes (IQR: 414–553.3), and the conversion rate was 18.3% (n=89). The rate of Clavien-Dindo grade ≥III complications was 31.4% (n=153), POPF was 21.2% (n=103), delayed gastric emptying was 15% (n=73), postpancreatectomy hemorrhage was 8.6% (n=42), and in-hospital/30-day mortality was 2.7% (n=13). Median length of stay was 12 days (IQR: 8–20). During simulation training, OSATS scores significantly improved (0.350 points/attempt (*ρ*), *P*<0.001) while the duration of reconstruction decreased (*P*<0.001). There was a significant decrease of the total number of errors made during consecutive attempts in the biotissue training (0.175 errors decrease per attempt, *P*=0.006]).

**Conclusions:**

We report on the first successfully completed international training program for RPD. Simulation established a standardized technical competency, although a learning-curve effect (POPF) remained detectable per center. These data highlight that safe adoption of complex procedures requires structured, team-based, proctored training beyond simulation alone.

Robotic pancreatoduodenectomy (RPD) aims to minimize the impact of this extensive surgical procedure and enhance patient recovery. The implementation of RPD into clinical practice comes with considerable challenges and an associated learning curve, leading to concerns about patient safety.^1^

Three randomized controlled trials (RCTs) have compared RPD versus open pancreatoduodenectomy (OPD). A single-center RCT from Heidelberg reported an RPD conversion rate of 23%, a Clavien-Dindo grade ≥3 rate of 36.9%, a postoperative pancreatic fistula (POPF) grade B/C rate of 26.9%, and a similar hospital stay between RPD and OPD. A multicenter RCT from China reported an RPD conversion rate of 2.4%, a POPF grade B/C rate of 8.9%, and a reduced time to functional recovery with RPD.^2^ The recent European DIPLOMA-2 RCT, conducted in centers that had surpassed the initial learning curve, showed similar complication rates, lower rates of POPF grade B/C, and a reduced time to functional recovery as compared with OPD.^3^

In the Dutch LAELAPS-3 program, based on the training program by the Pittsburgh group, the learning curve for achieving feasibility in RPD was 15 to 25 procedures, while mastery required ∼85 RPD procedures.^4,5^ Herein, the first 265 RPD procedures were performed with low mortality (90 d complication-related mortality 2.5%) and without an increased risk of major complications in the first and second phases of the learning curve, whereafter a post hoc analysis of 635 and 1000 RPD procedures showed increasingly improved results.^6,7^

Many large pancreatic centers worldwide are currently aiming to safely implement RPD. However, a European or international training program for RPD had not yet been established. Therefore, the European Consortium on Minimally Invasive Pancreatic Surgery (E-MIPS) implemented a multicenter training program for RPD (LEARNBOT) in European high-volume centers based on the Pittsburgh approach. This study aims to prospectively assess the impact of this training program on patient outcomes and learning curves. Furthermore, technical skills were analyzed during the training.

## METHODS

Data from all consecutive RPD procedures performed after the LEARNBOT training program were prospectively collected in the E-MIPS registry by each participating center and analyzed. The medical ethics review committee of Amsterdam UMC waived informed consent due to the observational nature of the study. The LEARNBOT training program was registered at the Dutch Trial Registry (NL8898).

### Eligibility

Patient eligibility for RPD included no vascular contact, no acute/chronic pancreatitis, and BMI <35 kg/m^2^. Center eligibility for training included an annual pancreatoduodenectomy (PD) volume of 50/center/year to allow for 20 RPD/center/year. The Miami guideline advises a minimum of 20 minimally invasive pancreatoduodenectomy (MIPD) procedures per center per year, since the literature shows that mortality is higher in MIPD than in OPD in the case of lower-volume strata.^7^ Given a proportion of 40% of patients who can undergo RPD, a center should have an annual volume of 50 PDs in order to fulfill this Miami guideline recommendation. Overall, 20 European centers were included. Per center, at least 2 surgeons were trained as a team. The team consisted of one robot-console surgeon and one tableside surgeon (ie, roles could switch as experience increased), without prior experience in RPD. Surgeons participating were required to have at least 5 years of experience with open/laparoscopic pancreatic surgery, as well as having performed a minimum of 10 robotic procedures, of which 3 were robotic left pancreatectomy procedures.^7^

### Training Program

All participating surgeons performed 26 da Vinci Skills Simulator exercises following the da Vinci Skills Simulator curriculum, of which 22/26 simulation exercises require a score >95%. Proof of this, that is, a photo, was shared with the LEARNBOT coordinator. After starting with this simulator curriculum, surgeons received the access codes for an online video databank describing multiple RPD procedures in great detail. This video databank was established by Prof. Melissa Hogg.^8^ Next, both participants were scheduled for external site (observer) visits to a proctor affiliated with the LEARNBOT training program, in which they observed an RPD performed and got a detailed step-by-step explanation from the proctor. This approach enabled surgeons to discuss the robotic approach to RPD in great detail with the proctor and to prepare for pitfalls by exchanging tips and tricks. At the time of implementation, proctorship required experience of >50 OPD cases, 100 RPD cases, and >5 years of robotic experience.

Hereafter, the trainer (LEARNBOT coordinator) visited the center for the on-site biotissue training. During the biotissue training, the surgeon had to perform at least 4 robotic pancreaticojejunostomies (PJs) and 4 robotic hepaticojejunostomies (HJs) (see Appendix 1: Table 1, Supplemental Digital Content 1, https://links.lww.com/SLA/F906). Artificial organs, that is, pancreas with soft and hard textures and small and large ducts, bile duct, and long double-layer small bowel, were provided by Heidelberg University.^9^ Robotic PJ was created using the modified Blumgart method, and a robotic hepaticojejunostomy was created using standing sutures (see Appendix 1, Supplemental Digital Content 1, https://links.lww.com/SLA/F906: Details of robot anastomoses). The materials were standardized for PDS for HJ and silk/vicryl for mattress sutures for PJ and PDS/prolene for duct-to-mucosa.

Ergonomic conditions were set according to the participants' needs before the start of the experiment and adjusted at any preferred moment by the participant. The biotissue training was recorded, and the videos of the biotissue training were first assessed through a “graduation video” before being allowed to perform RPD. The “graduation video” was scored by an expert RPD surgeon within 72 hours, who decided whether the surgeon could continue to the next step of the training program (RPD external site visit) or if more biotissue training was required, and this would be provided. All video recordings of the biotissue training were stored in high definition and were used for analysis. Finally, on-site (hands-on) proctoring was arranged with an experienced RPD proctor for the first RPD to proctor during the procedure (Supplementary Table 1, Supplemental Digital Content 1, https://links.lww.com/SLA/F906).

### Definitions

Operative time was defined as the time between the first incision and closure of the last incision. Conversion was defined as a switch to laparotomy to complete the procedure, other than specimen extraction. Postoperative complications were scored and classified using the Clavien-Dindo classification of surgical complications and were defined as major morbidity with grade 3 or higher.^10^ Body mass index (BMI) was calculated as weight in kg/m^2^. The definitions of the International Study Group for Pancreatic Surgery (ISGPS) and the International Study Group on Liver Surgery (ISGLS) were used to score POPF, postpancreatectomy hemorrhage (PPH), chyle leakage, delayed gastric emptying (DGE), and bile leakage.^11–15^ Surgical site infection (SSI) was classified according to the criteria established by the Centers for Disease Control and Prevention (CDC). Resection margins were categorized according to the Royal College of Pathologists definition and classified into R0 (distance margin to tumor ≥1 mm), R1 (distance margin to tumor <1 mm), and R2 (macroscopically positive margin).^16^ Complications requiring readmission and/or reoperation were recorded up to 30 and 90 days postoperatively.

### Data Collection

A survey (Google Survey, Mountain View, CA) was sent to all participants, inquiring about the current implementation of robotic surgery, annual surgical volumes (both for robot and total volume), and standards of care at the participating institution. This information was used for descriptions of (contra)indications and in the analyses. All clinical outcomes from RPD procedures performed after the LEARNBOT training program were collected in the ongoing E-MIPS registry, which collects data online (CASTOR) on MIPS procedures from participating European centers.

### Clinical Outcomes

Collected baseline characteristics included sex, age, BMI, comorbidity, medical history, and American Society of Anesthesiologists' physical status (ASA score). Intraoperative variables included operative time, blood loss, conversion, pancreatic texture, pancreatic duct diameter, and histopathology. Postoperative outcomes included the Clavien-Dindo complication rate, POPF, DGE, PPH, bile leakage, chyle leakage, incisional SSI, readmission, reoperation, hospital stay (day of surgery to discharge), in-hospital/30-day mortality, and 90-day mortality. Furthermore, histopathology tumor size, rate of R0/1 resections, number of malignant lymph nodes, and total number of retrieved nodes were collected.

### Statistical Analyses

Data were analyzed using IBM SPSS Statistics for Mac version 28.0 (IBM Corp., Armonk, NY) or R’s programming environment (version 4.5.3). A Student *t* test, Mann-Whitney *U* test, χ^2^ test, or Fisher exact test was used as suitable. Categorical data were presented as proportions, continuous data were presented as either mean and SD or median and interquartile range (IQR) as applicable. Alpha was set at a *P*<0.05; all analyses were 2-sided. Missing data were resolved by multiple imputation wherever appropriate. Regression analyses were performed using univariate univariable and multivariable regression analyses for dichotomous variables. All analyses were performed using R, version 4.5.0, with the marginaleffects (v0.30.0), mgcv (v1.9–3), and rms (v8.0–0) packages.

#### OSATS Analysis

The robotic biotissue HJ and PJ anastomoses were recorded and analyzed using Apple video software, Final Cut Pro. The procedure length and quality were scored using the Birkmeyer surgical performance grading.^17–19^ Videos of each anastomosis were edited and sent to 2 blinded graders. The videos were scored based on the objective structured assessment of technical skills (OSATS).^8,19,20^ Surgical video were further evaluated for technical performance and identified specific error categories during biotissue anastomoses, including (I) general technical errors: incorrect suture depth, insufficient knotting, unequal opening sizes, short suture threads, insufficient tightening, and small jejunum bites; (II) instrument-related errors: misuse of robotic instruments causing tissue tearing, excessive pulling, thread breakage, and needle bending/breaking; (III) visualization and orientation errors: suboptimal camera positioning and incorrect/insufficient use of the 3rd instrument; (IV) procedural errors: incorrect suture sequence and skipping protocol steps; (V) equipment failure: malfunctioning of thread or equipment (see appendix 2, Supplemental Digital Content 1, https://links.lww.com/SLA/F906). To assess possible continuous improvement with more training, the OSATS and errors were evaluated over the consecutive attempts.

#### Learning Curve Analysis

The case-mix adjusted learning curve was estimated using generalized additive mixed models in order to account for clustering of patients within centers, adjusted for a prespecified outcome-specific set of case-mix variables. Center experience (the consecutive number of patients that underwent RPD) was modeled as a continuous variable with restricted cubic splines (3 knots) to allow for a potentially nonlinear relationship between center experience and postoperative outcomes. The case-mix adjusted learning curve was subsequently estimated using regression standardization. The learning curves were reported as *P*_trend_ outcomes, wherein *P*_trend_<0.05 indicates a significant trend (*ρ*) toward an observed effect (such as faster operative time). *P*_trend_ were established for risk of conversion, operative duration, operative blood loss, risk of POPF, risk of DGE, risk of Clavien-Dindo ≥3, risk of reoperation, and risk of readmission.

In learning curve analysis, missing data were handled using flexible multiple imputation models, including the exposure variable (the center experience variable), the outcome variables, and all case-mix variables (age, sex, BMI, ASA, texture, pancreatic duct size, PDAC vs. non-PDAC disease, and tumor size). Continuous variables were modeled in the imputation and analysis model using restricted cubic splines. In total, 30 imputed data sets were generated after 50 burn-in iterations. Learning curves were pooled on the link scale across multiply imputed data sets using the Rubin rules.

## RESULTS

### Center and Patient Characteristics

A total of 486 RPD procedures were performed by 40 trained surgeons from 20 high-volume centers in 12 countries who had completed the training program; this involved a median of 36 RPD procedures per center (range: 2–101 procedures). All surgeons’ graduation videos were OSATS above >3 and approved by the expert RPD surgeon. Experience with pancreatic resections ranged from 6 to >20 years. All centers were proctored for their first RPD by an affiliated experienced RPD surgeon from the LEARNBOT group. Median age of patients was 70 years (IQR: 62–76), and 47% of patients were female. The majority of patients were ASA II (60.9%). Previous abdominal surgery had been performed in 146 patients (30%). Any comorbidity was present in 65.2% of the patients (Table S2 Supplemental Digital Content 1, https://links.lww.com/SLA/F973).

### Intraoperative Outcome

Among 486 who underwent RPD, the median operative time was 475 minutes (IQR: 414–553), and the median estimated blood loss was 200 mL (IQR: 100–400) with an 18.3% (n=89) conversion rate. Pancreatic texture was soft in 56.2% of patients (n=273). The median pancreatic duct diameter was 3.8 mm (IQR: 2–5), and somatostatin analog was administered in 27.8% (n=135). Histopathologic analysis revealed that the most common diagnosis was pancreatic ductal adenocarcinoma (35.8%, n=174) (Table S3 Supplemental Digital Content 1, https://links.lww.com/SLA/F973).

### Postoperative Outcome

The rate of major complications was 31.5% (n=153), POPF 21.2% (n=103), DGE 15.0% (n=73), PPH 8.6% (n=42), bile leakage 7.8% (n=38), and chyle leakage 1.6% (n=8). Incisional SSIs (SSI 1 and 2) occurred in 6.0% (n=29) of patients, with 3.1% (n=15) classified as superficial and 14 cases (2.9%) as deep incisional.

The median initial hospital stay was 12 days (IQR: 8–20), and 14.8% (n=72) of patients were readmitted. Reoperation was necessary in 14.4% (n=70), while the in-hospital/30-day mortality rate was 2.7% (n=13) and the 90-day mortality was 3.5% (n=17). For patients with pancreatic ductal adenocarcinoma (PDAC), an R0 resection (distance to all margins and specimen surfaces ≥1 mm) was achieved in 47.1% (n=82). The median number of lymph nodes examined was 20 (IQR: 14–26). The median maximum tumor diameter was 27 mm (IQR: 21–35) (Table S4 Supplemental Digital Content 1, https://links.lww.com/SLA/F973).

### Intra- and Postoperative Outcome Over Time

Comparison of the first 15 RPD procedures performed per center with subsequent procedures demonstrated a reduction in operative time with increasing experience (485 vs. 464 min, *P*=0.004). Reported blood loss was higher over time [200 (IQR: 50–300) vs. 200 (IQR: 100–500) mL, *P*<0.001]. Conversion to open surgery remained comparable (17.1% vs. 19.3%, *P*=0.532). The rate of major complications (Clavien-Dindo ≥3) did not significantly differ after the first 15 procedures per center (33.3% vs. 29.9%, *P*=0.420). No significant differences were observed in POPF grade B/C, DGE B/C, PPH grade B/C, chyle leak grade B/C, length of hospital stay, readmission, reoperation, or mortality. In contrast, higher rates of bile leakage grade B/C were observed in later cases (Table 1).

**TABLE 1:** Intra- and Postoperative Outcomes Stratified for Experience Per Center (First 15 RPD vs. Thereafter)

	First 15 RPD per Center RPD (n=222), n (%) or Median [IQR]	Beyond 15 RPD per Center RPD (n=264), n (%) or Median [IQR]	*P*
Intraoperative outcomes			
Operative time (min)	485 [IQR: 425–582]	464 [IQR: 408–530]	0.004
Blood loss (mL)	200 [IQR: 50–300]	200 [IQR: 100–500]	<0.001
Conversion to open	38 (17.1)	51 (19.3)	0.532
Pancreatic texture			
Soft	125 (56.3)	148 (56.1)	0.248
Firm	70 (31.5)	104 (39.4)	
Unknown	27 (12.2)	12 (4.5)	
Pancreatic duct diameter (mm)	3 [2–5]	4 [2–6]	0.184
Pancreatic ductal adenocarcinoma	68 (30.6)	106 (40.2)	0.029
Postoperative outcomes			
Clavien-Dindo complication grade ≥3	74 (33.3)	79 (29.9)	0.420
Postoperative pancreatic fistula (B/C)	48 (21.6)	55 (20.8)	0.812
Grade C	8 (3.6)	13 (4.9)	
Delayed gastric emptying (B/C)	31 (14)	42 (15.9)	0.552
Grade C	13 (5.9)	12 (4.5)	
Postpancreatectomy hemorrhage (B/C)	18 (8.1)	24 (9.1)	0.724
Grade C	10 (4.5)	11 (4.2)	
Bile leakage (B/C)	9 (4.1)	29 (11.0)	0.005
Grade C	2 (0.9)	9 (3.4)	
Chyle leakage (B/C)	4 (1.8)	4 (1.5)	0.8
Grade C	0	0	
Length of initial hospital stay (d)	12 [8–22]	11 [8–19]	0.968
Readmission within 30 d	31 (14.0)	41 (15.5)	0.152
90 d reoperation	38 (17.1)	32 (12.1)	0.419
In-hospital/30 d mortality	7 (3.2)	6 (2.3)	0.458
Mortality 90 d	9 (4.1)	8 (3.0)	0.541

% indicates percentage; IQR, interquartile range; n, number of patients; RPD, robotic pancreatoduodenectomy.

### Learning Curve CUSUM

For intraoperative outcomes, CUSUM analysis demonstrated a significant upward trend in operative blood loss (*P*_trend_<0.0001), whereas operative duration demonstrated a significant downward trend with increasing experience (*P*_trend_=0.0002). No significant trends were observed for conversion to open surgery (Fig. 1). For postoperative outcomes, CUSUM analysis demonstrated a significant downward trend in the predicted risk of POPF with increasing center experience (*P*_trend_=0.043). No significant trends were observed for major complications (Clavien-Dindo ≥3), DGE, readmission, or reoperation, with these outcomes remaining relatively stable as experience increased (*P*_trend_=0.49, *P*_trend_=0.71, *P*_trend_=0.62, and *P*_trend_=0.41, respectively) (Fig. 2).

**FIGURE 1:**
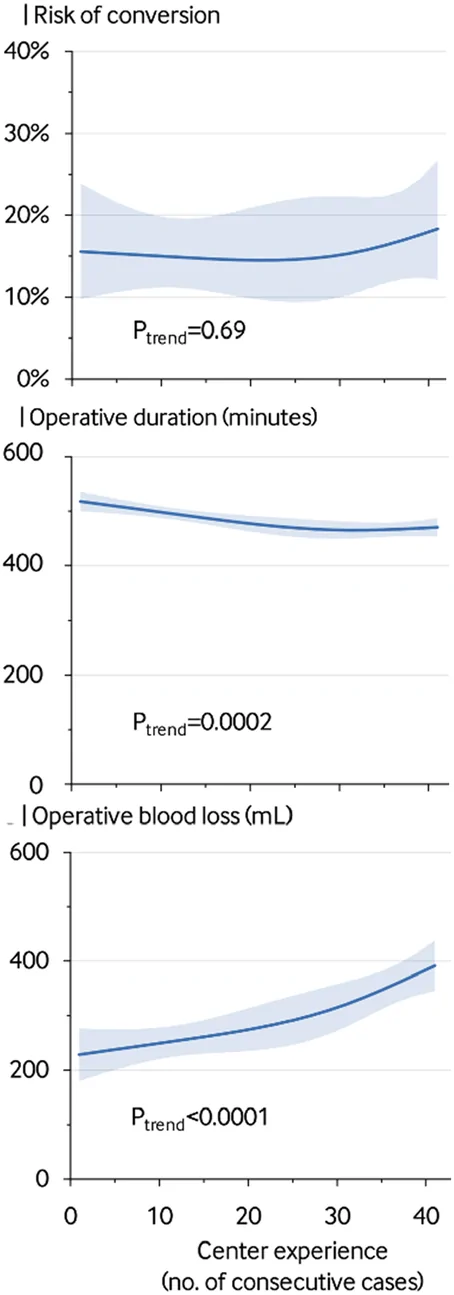
Intraoperative (feasibility) learning curve for robotic pancreatoduodenectomy, risk-adjusted.

**FIGURE 2:**
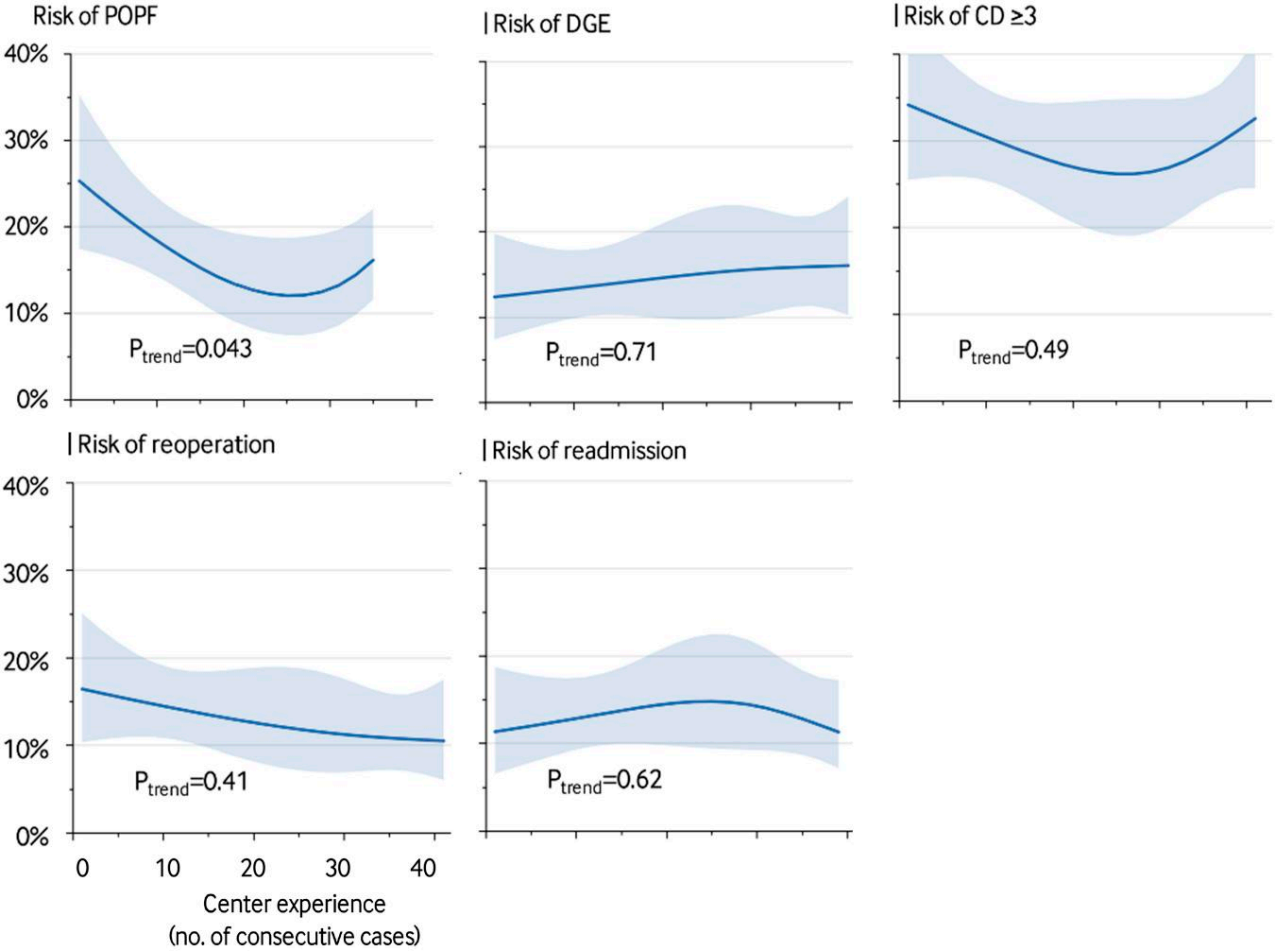
Intraoperative (feasibility) learning curve for robotic pancreatoduodenectomy, risk-adjusted.

### OSATS and Errors During Biotissue Training: Safety and Potential Improvement

The OSATS scores (attainable 1–5) were a median of 3.7 (IQR: 3.2–4.2) for PJs and 4.1 (IQR: 3.4–4.4) for HJs, indicating an above-average performance. During simulation training, OSATS scores significantly improved [0.350 OSATS points per attempt (*ρ*=0.350), *P*<0.001], and the duration of reconstruction decreased significantly (21 min per attempt, *P*<0.001). All OSATS elements were ≥3 grades on the last attempt. The OSATS median for PJ at the final attempt was 3.7 (IQR: 3.1–4.1), and the OSATS median for HJ at the final attempt was 4.1 (IQR: 3.4–4.4). There was a significant decrease in the total number of errors made during consecutive attempts [0.175 errors decrease per attempt (*ρ*=−0.175, *P*=0.006)], although the median number of errors made in the last attempt of the HJ was 2.0 (IQR: 0.75–3.0), and for the PJ it was 6.0 (IQR: 3–9) (Fig. 3).

**FIGURE 3:**
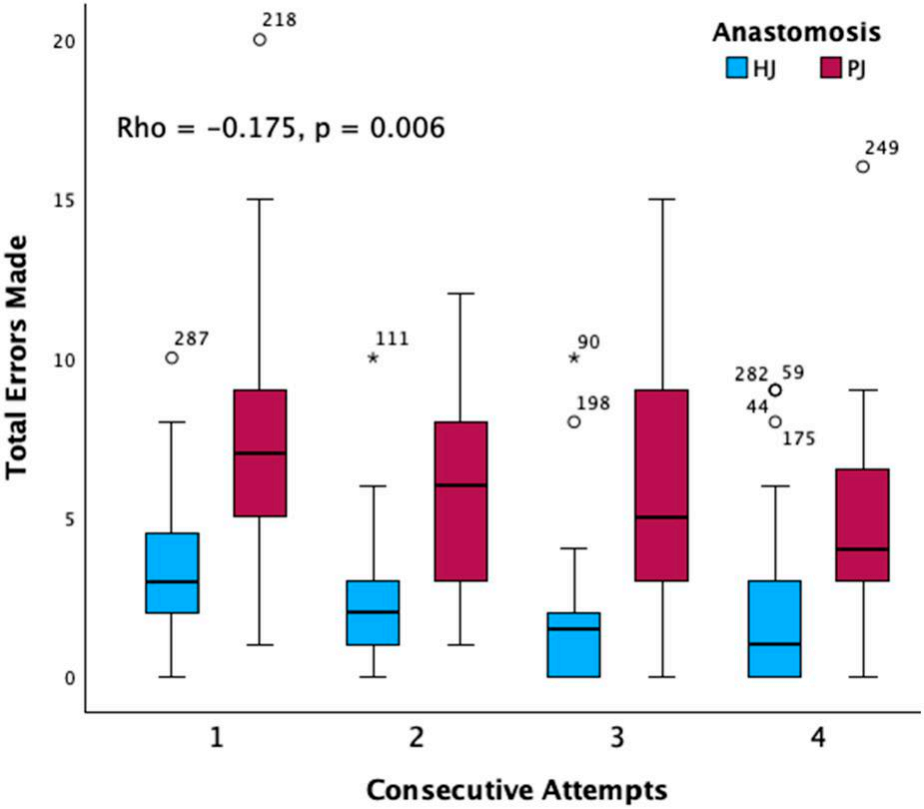
Boxplot of errors made across 4 consecutive robotic HJ and PJ anastomoses.

### Risk Factors for Conversion

Male sex was more frequent in the conversion group, although this difference was not statistically significant (55.7% vs. 51.4%, *P*=0.371). Similarly, certain centers demonstrated a disproportionately high number of conversions. In contrast, ASA class III was significantly more common among converted cases (40.1% vs. 25.1%, *P*=0.019). A multivariable analysis aimed to identify risk factors for conversion and revealed that an increase in intraoperative pancreatic duct diameter correlated with a lower risk of conversion: odds ratio of 0.86, 95% CI: 0.753–0.973, indicating that each 1 mm increase in duct diameter showed a relative risk reduction in conversion of 14.4%. Patients with a soft/normal pancreatic texture had a significantly higher risk of conversion compared with those with a firm pancreas (OR: 3.24, 95% CI: 1.78–5.92).

## DISCUSSION

This first report of a European training program in RPD (LEARNBOT) demonstrates its feasibility and safety when expanding RPD to high-volume centers through a structured curriculum. In this early learning curve cohort of 20 teams across 12 countries, we observed acceptable outcomes, including 31.5% Clavien-Dindo grade ≥3 complications and a 2.7% in-hospital/30-day mortality rate. Importantly, while the primary focus of this feasibility study was perioperative safety, the oncological adequacy of the procedures was maintained. R0 resection rates and lymph node yields were comparable to established benchmarks for open surgery, suggesting that the learning curve does not come at the cost of oncological radicality. OSATS scores were at and above international expert reports for RPD. Although a learning curve effect remained, risk-adjusted learning curve analysis showed only a downward trend of POPF. While a long learning curve for the PJ is reported, after 20 cases in the LEARNBOT cohort, the risk of POPF was lower than 15% to 20%. These findings suggest that a standardized, simulation-based training pathway can mitigate the historical risks associated with the early adoption of minimally invasive pancreatic surgery.

The outcomes of the present cohort are largely comparable to the preceding Dutch LAELAPS-3 training program, which was also based on the Pittsburgh approach.^21^ The rate of severe complications (31.5%) was lower than that reported in the LAELAPS-3 cohort (44.4%), while 30-day mortality rates were comparable (2.7% vs. 2.5%).^21^ These results are also largely consistent with high-volume benchmarks for OPD and the robotic experience at mature centers like UPMC.^22^ Still, there is room for improvement, as the conversion rate in this series was quite high at 18.3%, clearly higher than the 6.5% in the LAELAPS-3^5^ cohort. In centers with higher conversion rates, we did not observe a corresponding increase in mortality or severe morbidity. Several participating surgeons maintained a very low threshold for conversion when technical difficulties or progression stalls occurred, prioritizing patient safety over the completion of a minimally invasive procedure during their learning curve. A longer follow-up is required to assess whether the conversion rates will decrease to levels as seen in international literature.^3,5,22^

A unique aspect of this study was the “graduation” of simulation performance using evaluation videos of biotissue anastomoses. Birkmeyer et al validated OSATS scores during video grading, and van den Broek et al and Zwart et al validated the use of video grading in a post hoc analysis of RPD procedures after the LAELAPS-3 training.^19,20,23–25^ Still, one of the main findings in this report was a residual risk of postoperative anastomotic complications that might be explained by surgical errors not incorporated in the OSATS score. OSATS scores on biotissue models improved significantly during training and reached above-average performance for all elements at final attempts. However, the median number of errors made in the last attempt of the PJ was 6.0, with a relatively low decrease per attempt. This could indicate that more training and experience are required to improve error rates at the final attempt. The latter seems likely, as a residual learning curve for POPF was shown in the risk-adjusted CUSUM analysis. Nevertheless, the exact added value of the LEARNBOT training program remains unknown, as we did not include a control group without training. Only an RCT could determine this, but this would clearly be highly challenging. Ultimately, in the future, the LEARNBOT cohort could grow into a sample size that allows for risk-factor analysis between scored surgical performance and clinical outcomes. Furthermore, focus on training efficiency is particularly relevant in the broader economic context of RPD implementation. While the adoption of robotic surgery is frequently scrutinized for higher initial costs compared with laparoscopic or open approaches, a high-volume center demonstrated that as surgical proficiency improves and outcomes are optimized, the total costs of RPD decrease significantly, eventually achieving parity with OPD.^26^ Consequently, by accelerating technical acquisition and mitigating early-phase complications, structured training programs may contribute to cost-effectiveness while maintaining patient safety.

As reported by van Hilst et al^27^ in 2017, the absence of an international (European) training program for RPD represented a deficit in surgical education, particularly given the safety concerns associated with the early learning curve of RPD. As the early termination of the LEOPARD-2 trial demonstrated,^28^ the transition to minimally invasive techniques carries inherent risks if the learning curve is not managed through rigorous preparation and adequate volume.^29^ Unlike open surgery, where tactile feedback and direct visualization may partially compensate for technical inexperience, RPD requires the mastery of complex hand-eye coordination and robotic instrumentation without these sensory aids.^30,31^ Consequently, the reliance on the traditional apprenticeship model is insufficient for such complex procedures.^32,33^ Structured training (eg, with biotissue simulation and proctoring) is recommended to close the critical bridge between preclinical competence and proficiency.^7^ The matured high-fidelity 3D-printed models allow surgeons to perfect the most unforgiving steps of the procedure—specifically the pancreatic anastomosis—in a realistic and risk-free environment,^9^ thereby reducing the likelihood of intraoperative errors during the initial adoption phase. Empirical evidence supports this approach; a post hoc analysis of the LAELAPS-3 program confirmed that centers adhering to a structured curriculum achieved significantly shorter learning curves compared with pioneering institutions.^23^ This underscores the capacity of simulation to accelerate technical acquisition and enhance patient safety. Data from mature high-volume centers further validate the efficacy of structured implementation. The UPMC reported excellent long-term outcomes over a decade of 500 consecutive procedures, achieving a Clavien-Dindo grade ≥3 complication rate of 24.8% and a POPF grade B/C rate of 7.8%.^22^ Furthermore, the sustained improvement observed in the follow-up of the LAELAPS-3 trial indicates that skills acquired through structured training are both durable and scalable across multiple institutions.^5^ The LEARNBOT experience demonstrates that the benefits of RPD can be safely extended to a broad network of centers through a standardized, simulation-based curriculum. These findings suggest that structured training should no longer be viewed as optional but rather as a prerequisite for the ethical introduction of complex surgical innovations. By decoupling the learning curve from patient outcomes, such programs ensure that the adoption of robotic surgery is driven by safety and quality.^5^ Still, the current study report can only assess the feasibility and safety of the LEARNBOT training program for RPD but cannot claim any superiority over other methods.

Multiple RCTs comparing MIPD and OPD were conducted. A single-center phase 2 RCT from Heidelberg (the EUROPA trial) reported higher complication rates, similar hospital stay, higher hospital costs, and a 23% conversion rate to OPD.^28^ A multicenter phase 3 RCT from Beijing, China, reported a shorter hospital stay after RPD and similar complication rates compared with OPD.^2^ The recent European DIPLOMA-2 RCT conducted in centers that had surpassed the initial learning curve showed similar complication rates, lower rates of pancreatic fistula, and reduced time to functional recovery as compared with OPD.^3^

Several limitations must also be acknowledged. First, despite the prospective design, the study remains observational and subject to selection bias, as favorable candidates were likely selected for the initial learning curve cases. Second, the conversion rate of 18.3% was higher than that observed in mature programs. However, this appears to reflect a low threshold for conversion rather than compromised safety. It is also important to note that the patients who are technically easiest to resect are often those at highest risk of postoperative complications, particularly those with small tumors and a soft pancreas. These patients are most likely to be selected as the initial robotic cases at each center. Third, this paper reports on the first outcomes after the training program, meaning that centers did not surpass their learning curve yet. This will be analyzed in future research aiming for 50 to 100 postoperative procedures per center. The outcomes in the paper report on feasibility and safety. Fourth, although the LEARNBOT methodology improved upon the LAELAPS-3 design by utilizing validated biotissue models as a strict preclinical “gatekeeper,” a limitation of the current study is the scope of the technical skills analysis. The data presented here are restricted to aggregate proficiency scores, without a detailed breakdown of individual OSATS domains or specific intraoperative “error types.” Consequently, while the program ensured a verified baseline of competence, the specific technical nuances and error patterns characterizing the early learning curve were not assessed in this report and remain the subject of planned future investigations. Fifth, there was no control group since all centers were trained. A randomized study would have been optimal but would be highly challenging in this setting. Potentially, a “stepped wedge” randomized trial design or comparisons against matched historical registry data would be feasible methodologies to further validate the program’s specific impact. Main strengths of this study include the first pan-European training program for RPD using data from the prospective E-MIPS registry, ensuring rigorous data capture and minimizing the retrospective reporting bias often seen in surgical learning curve analyses.

In conclusion, this first international multicenter training program demonstrates that expanding RPD to high-volume centers is feasible and safe when governed by a structured, simulation-based curriculum. By adhering to a rigorous “one team per center” training model, participating institutions achieved perioperative outcomes comparable to established benchmarks, even during the early learning curve. This suggests that the curriculum successfully established a protective baseline toward proficiency and protected patients from high rates of POPF associated with the learning curve of RPD.

## Supplementary Material

## DISCUSSANTS

### Moritz Schmelzle (Hannover, Germany)

I’m happy to serve as the first discussant on this great paper. First of all, I would like to congratulate you, your co-authors, and all participating centers for their commitment and for conducting a scientific evaluation of robotic training in this highly complex field of pancreatic surgery. As you stated, the study confirms that the implementation of an advanced robotic training program across European centers is feasible. From my personal perspective, this alone represents a relevant contribution, particularly in the context of the further development of a European surgical training curriculum. Moreover, the prospective collection of data is especially valuable in a field that, from a clinical perspective, remains at a relatively early stage of development. During simulation training, as you pointed out, the OSAT scores improved significantly while reconstruction time decreased. However, the central research question of this study extends beyond performance in the dry lab. The underlying hypothesis was that the structured training program and the learning curve achieved during simulation would positively influence patient outcomes.

So, I have 3 questions for you. First, can we really rule out that comparable postoperative outcomes would have been achieved without the LEARNBOT curriculum, particularly in the absence of a randomized training versus no training comparison? Second, you reported on the subjective participant satisfaction and also the improved OSAT scores. This is something that we routinely see in such controlled programs, but from your perspective, what is the measurable clinical added value of this program, especially with regard to the patient outcomes? Third, I think this is quite debatable, but from your perspective, how can we identify at least components of the curriculum that are truly high-yield, and do these benefits justify the considerable logistical and financial costs of implementing such a program?

### Response From Leia Jones (Amsterdam, The Netherlands)

That is an important point. Regarding your first question, based on the current results, we cannot draw such a conclusion because the study did not include a control group. Addressing this question would require a randomized trial.

As more data will become available, we plan to perform further analyses by comparing outcomes from centers participating in the training program with those from centers within the EMIPS registry that did not implement such a program.

Regarding your second question, in my view, the main benefit of the program is that it shifts the learning phase from the intraoperative setting, where surgeons learn while operating on patients, to a preoperative training environment. This is supported by the observed improvements in OSATS scores, reductions in technical errors, and shorter reconstruction times over the course of training.

Therefore, I believe that transferring the learning curve to the preoperative setting has the potential to improve patient safety and ultimately improve patient outcomes.

Regarding your third question on costs, training involved a dedicated PhD researcher traveling to the participating centers for 2 days. Having personally conducted these training sessions, I believe that this approach is less costly than going through the learning curve during live operative procedures and having the costs of an OR and surgical team involved. Nevertheless, a formal cost analysis would be required to substantiate this assumption.

Furthermore, additional analyses focusing on technical errors and OSATS performance are warranted. Although we observed reductions in reconstruction time and improvements in technical performance, no clear effect was evident on clinical outcomes during the early phase of the learning curve. Such effects may become apparent with longer follow-up and the inclusion of additional procedures.

### Mickaël Lesurtel (Clichy, France)

Congratulations on this outstanding effort to standardize the training for such difficult surgery. From a technical point of view, I know that in Amsterdam, usually 2 senior surgeons are involved, one at the console, the other one at the operating table. Therefore, I would like to know how you standardize the surgical technique?

### Response From Leia Jones (Amsterdam, The Netherlands)

The surgical technique was standardized according to the established approach used at UPMC Pittsburgh, which trained us in Amsterdam UMC. Specifically, procedures were performed with both a console surgeon and a tableside surgeon. For the pancreaticojejunostomy, a modified Blumgart technique was standardized, whereas interrupted sutures were used for the hepaticojejunostomy.
