# Supplementary material for: Pan-European Training Program for Robotic Pancreatoduodenectomy (LEARNBOT): Prospective Multicenter Study on Feasibility and Outcome
Source: Ann Surg. 2026 Oct 7;284(5):993–1001. doi: 10.1097/SLA.0000000000007170 (PMC13652116; doi:10.1097/SLA.0000000000007170)
Supplement: Supplementary file 1 [file sla-284-0993-s001.docx]

# Supplementary material

## Supplementary table 1. LEARNBOT training program steps and details of robot anastomoses

### LEARNBOT training program for robot pancreatoduodenectomy consisting of 6 steps

| LEARNBOT program | | |
| --- | --- | --- |
| 1 | Pre-training simulator  (LEARNBOT coordinated) | - da Vinci® Simulator curriculum* - 22/26 simulation exercises, score >95% required |
| 2 | Online video assessment  (LEARNBOT coordinated) | - Online video library with multiple robot pancreatoduodenectomy procedures - At least 4 hours |
| 3 | Day 1 Robot biotissue  (Trainer visits local center) | - Debriefing simulator and video - Biotissue drills: PJ and HJ - See Appendix: details of robotic anastomosis |
| 4 | Day 2 Robot biotissue  (Trainer visits local center) | - Biotissue drills: PJ and HJ - Planning external site visit and onsite proctoring |
| 5 | Robot pancreatoduodenectomy external site visit  (LEARNBOT coordinated) | Visit a robot pancreatoduodenectomy (Orleans, Pisa, Utrecht, Rotterdam, Brescia, Amsterdam, Heidelberg) |
| 6 | Robot pancreatoduodenectomy on site proctoring  (Trainer and proctor visit local center) | Via regular da Vinci® proctor program |

### Details of robot anastomoses

#### Hepaticojejunostomy

**One hepaticojejunostomy is created using the following procedure comprising eight standing sutures.**

**Step 1. At the 6 O’clock position, a** suture 5-0 14CM 13-17MM RB-1 Needle is driven from inside the bile duct lumen through the outside of the jejunum. A double knot is created followed by 3 loop knots. Scissors are used to cut of the suture at the resulting standing knot. The leftover material is used in the standing created at step 2.

**Step 2. At the half past 4 position, the leftover** suture 5-0 14CM 13-17MM RB-1 Needle from step 1 is driven from inside the bile duct lumen through the outside of the jejunum. A double knot is created followed by 3 loop knots. Scissors are used to cut of the suture at the resulting standing knot. The needle is placed on the right-hand corner of the set-up and extruded with the help of the assistant.

**Step 3. At the half past 7 position, a** suture 5-0 14CM 13-17MM RB-1 Needle is driven from inside the bile duct lumen through the outside of the jejunum. A double knot is created followed by 3 loop knots. Scissors are used to cut of the suture at the resulting standing knot. The leftover material is used in the standing knot created at step 4.

**Step 4. At the 3 O’clock position, the leftover** suture 5-0 14CM 13-17MM RB-1 Needle from step 3 is driven from outside of the bile duct lumen through the inside of the jejunum. A double knot is created followed by 3 loop knots. Scissors are used to cut of the suture at the resulting standing knot. The needle is placed on the right-hand corner of the set-up and extruded with the help of the assistant.

**Step 5. At the 9 O’clock position, a** suture 5-0 14CM 13-17MM RB-1 Needle is driven from outside the bile duct through the inside of the jejunum. A double knot is created followed by 3 loop knots. Scissors are used to cut of the suture at the resulting standing knot. The leftover material is used in the standing knot created at step 6.

**Step 6. At the half past 1 position, a** suture 5-0 14CM 17MM RB-1 Needle from step 5 is driven from outside the bile duct lumen through the outside of the jejunum. A double knot is created followed by 3 loop knots. Scissors are used to cut of the suture at the resulting standing knot. The needle is placed on the right-hand corner of the set-up and extruded with the help of the assistant.

**Step 7. At the half past 10 position, the leftover** suture 5-0 14CM 13-17MM RB-1 Needle is driven from outside of the bile duct lumen through the inside of the jejunum. A double knot is created followed by 3 loop knots. Scissors are used to cut of the suture at the resulting standing knot. The leftover material is used in the standing knot created at step 8

**Step 8. At the 12 O’clock position, a** suture 5-0 14CM 13-17MM RB-1 Needle from step 7 is driven from outside the bile duct lumen through the outside of the jejunum. A double knot is created followed by 3 loop knots. Scissors are used to cut of the suture at the resulting standing knot. The needle is placed on the right-hand corner of the set-up and extruded with the help of the assistant.

#### Pancreaticojejunostomy

**One pancreaticojejunostomy is created using the following modified Blumgart method.**^14^

**Step 1.**

**Step 1.a. At the cranial position of the pancreas, denoted with 1., a** suture 2-0 23CM 36MM CT-1 needle is driven through the anterior plane of the pancreas to the posterior plane. The needle is then driven through the jejunum in a cranial to caudal direction and back through the pancreas from posterior to anterior to create a mattress stitch. The suture is tightened, a double knot is tied and followed by two loops. The needle is then placed out of the working field.

**Step 1.b. At the central position of the pancreas, just cranial of the pancreatic duct, denoted with 2., a** suture 2-0 23CM 36MM CT-1 needle is driven through the anterior plane of the pancreas to the posterior plane. The needle is then driven through the jejunum in a cranial to caudal direction (it is important to take a big bite of jejunum here to minimize the risk of narrowing the pancreatic duct) and back through the pancreas, just caudal from the pancreatic duct, from posterior to anterior to create a mattress stitch. A stent is inserted in the pancreatic duct. The suture is tightened, a double knot is tied and followed by three loops. The needle is then placed out of the working field.

**Step 1.c. At the caudal position of the pancreas, denoted with 3., a** suture 2-0 23CM 36MM CT-1 needle is driven through the anterior plane of the pancreas to the posterior plane. The needle is then driven through the jejunum in a cranial to caudal direction and back through the pancreas from posterior to anterior to create a mattress stitch. The suture is tightened, a double knot is tied and followed by three loops. The needle is then placed out of the working field.

**Step 2.**

**Step 2.a.** The ductal stent is removed from the pancreatic duct and extracted by the assistant.

**Step 2.b. At the ‘legs of the star’, t**he first posterior duct-to-mucosa stitches are created **with a** 5-0 <14CM 13-17MM RB-1 Needle from inside the pancreatic duct lumen through the outside of the jejunum. A double knot is created followed by 3 loop knots. Scissors are used to cut off the suture at the resulting standing knot. The leftover material is used in the succeeding standing stitch created or the needle is placed on the right-hand corner of the set-up and extruded with the help of the assistant.

**Step 2.c.** The stent is handed by the assistant. The ductal stent is inserted in the pancreatic duct and in the jejunum.

**Step 2.d.** The first and second anterior duct-to-mucosa stitches are created **at the ‘arms of a star’ position, a** suture 5-0 <14CM 13-17MM RB-1 Needle is driven from outside of the jejunum through the inside of the pancreatic duct lumen. A double knot is created followed by 3 loop knots. Scissors are used to cut off the suture at the resulting standing knot. The leftover material is used in the succeeding standing stitch created.

**At the ‘head of the star’ position, the leftover** suture 5-0 <14CM 13-17MM RB-1 Needle from step 2.d. is driven from outside of the jejunum through the inside pancreatic duct. A double knot is created followed by 3 loop knots. Scissors are used to cut off the suture at the resulting standing knot. The needle is placed on the right-hand corner of the set-up and extruded with the help of the assistant.

**Step 3.**

**Step 3.a. At the cranial position of the pancreas, denoted with 1., the** suture 2-0 23CM 36MM CT-1 needle is driven through the jejunum in a cranial to caudal direction, just laterally from the knot created at step 1.a.. The suture is tightened, a double knot is tied and followed by three loops. Scissors are used to cut off the suture at the resulting knot and the leftover material is placed outside of the working field or handed directly to the assistant.

**Step 3.b. At the central position of the pancreas, denoted with 2., the**  suture 2-0 23CM 36MM CT-1 needle is driven through the jejunum in a cranial to caudal direction, just laterally from the knot created at step 2.a.. The suture is tightened, a double knot is tied and followed by three loops. Scissors are used to cut off the suture at the resulting knot and the leftover material is placed outside of the working field or handed directly to the assistant.

**Step 3.c. At the caudal position of the pancreas, denoted with 3., the** suture 2-0 23CM 36MM CT-1 needle is driven through the jejunum in a cranial to caudal direction, just laterally from the knot created at step 3.a.. The suture is tightened, a double knot is tied and followed by three loops. Scissors are used to cut off the suture at the resulting knot and the leftover material is placed outside of the working field or handed directly to the assistant.

Supplementary appendix 1. Details of robot anastomoses

## Appendix 2. The video assessment for safety and surgical skill

*Table 1. Error grading tool; counted as total*

| **Error Category** | **Description** |  |
| --- | --- | --- |
| **General technical errors** | Incorrect suture depth, insufficient knots/loops, attempt at stitch with too short suture thread, insufficient tightening (air-knot), small jejunum bites, sloppy enterotomy. |  |
| **Instrument-related errors** | Misuse of robotic instruments causing tissue tearing, excessive pulling, thread breakage, needle bending/breaking, careless with needle. |  |
| **Visualization and orientation errors** | Incorrect/insufficient use of 3^rd^ arm and camera for optimal visualization of the biotissue |  |
| **Procedural errors** | Incorrect suture sequence, skipping protocol steps, incorrect direction of stitch (Mattress/DTM/Buttress) |  |
| **Equipment failure** | Malfunctioning of thread or equipment. |  |
| DTM: Duct-To-Mucosa | | |

*Table 2. Modified OSATS framework; total score of 5*

| **Grading Definition** | **Description** |
| --- | --- |
| **1** | Deficient/Traumatic |
| **2** | Lacking/Lacks finesse |
| **3** | Average |
| **4** | Skilled |
| **5** | Master/Flawless |
|  |  |
| ***Grading Aspects*** | ***Description*** |
| **Gentleness** | Gentle tissue handling that does not result in injury |
| **Time and Motion** | Economy of motion, maximum efficiency |
| **Instrument Handling** | Fluid use of instruments without awkwardness |
| **Tissue Exposure** | Retraction that allows for good visualization and proper tissue alignment |
| **Flow of Operation** | Smooth transitions from one part of the operation to another |
| **Summary Score** | Overall assessment of technical skill |
