# Supplementary material for: Pan-European Training Program for Robotic Pancreatoduodenectomy (LEARNBOT): Prospective Multicenter Study on Feasibility and Outcome
Source: Ann Surg. 2026 Oct 7;284(5):993–1001. doi: 10.1097/SLA.0000000000007170 (PMC13652116; doi:10.1097/SLA.0000000000007170)
Supplement: Supplementary file 2 [file sla-284-0993-s002.docx]

## Table 1. Baseline characteristics

|  | RPD (n=486) |
| --- | --- |
| Age, median [IQR] | 70 [62-76] |
| BMI, kg/m^2^, median [IQR] | 24.6 [22.2-27.5] |
| Female, n (%) | 229 (47.1) |
| ASA status, n (%)  I  II  III  Unknown | 36 (7.4)  296 (60.9)  147 (30.2)  7 (1.4) |
| Previous abdominal surgery, n (%) | 146 (30) |
| ****Any comorbidity, n (%)****  **Myocard infarct**  **Diabetes, insulin dependent**  CVA | 317 (65.2)  23 (4.7)  47 (9.7)  27 (5.6) |
| Neoadjuvant chemo (radio)therapy, n (%)  Chemoradiation therapy  Chemotherapy | 5 (1)  14 (2.9) |

**Table legend**: RPD = robotic pancreatoduodenectomy; n = number of patients; IQR = interquartile range; BMI = body mass index; kg = kilograms; m2 = square meters; ASA = American Society of Anesthesiologists; CVA = cerebrovascular accident; % = percentage.

## Table 2. Intra-operative outcome

|  | RPD (n=486) |
| --- | --- |
| Operative time, min., median [IQR] | 475 [414-553.3] |
| Blood loss, mL, median [IQR] | 200 [100-400] |
| Conversion to open, n (%) | 89 (18.3%) |
| Pancreatic texture  Soft  Firm  Unknown | 273 (56.2)  174 (35.8)  39 (8.1) |
| Pancreatic duct diameter, mm, median (IQR) | 3.8 [2-5] |
| Somatostatin used | 135 (27.8) |
| Histopathology  Pancreatic ductal adenocarcinoma  Distal cholangiocarcinoma  Ampullary cancer  Duodenum adenocarcinoma  pNET  IPMN  MCN  Adenoma  SPN  SCN  Chronic pancreatitis  Chronic cholangitis  Other  Unknown | 174 (35.8)  44 (9.1)  56 (11.5)  8 (1.6)  33 (6.8)  45 (9.3)  3 (0.6)  27 (5.6)  2 (0.4)  4 (0.8)  9 (1.9)  2 (0.4)  56 (11.5)  3 (0.6) |

**Table legend**: RPD = robotic pancreatoduodenectomy; n = number of patients; min. = minutes; IQR = interquartile range; mL = milliliters; mm = millimeters; pNET = pancreatic neuroendocrine tumor; IPMN = intraductal papillary mucinous neoplasm; MCN = mucinous cystic neoplasm; SPN = solid pseudopapillary neoplasm; SCN = serous cystic neoplasm.

## Table 3. Postoperative surgical and oncological outcome

|  | RPD (n=486) |
| --- | --- |
| **Clavien-Dindo complication grade ≥ 3, n (%)** | 153 (31.5) |
| Postoperative pancreatic fistula (B/C), n (%)  **Grade C** | 103 (21.2)  21 (4.3) |
| Delayed gastric emptying (B/C), n (%)  Grade C | 73 (15.0)  25 (5.1) |
| Postpancreatectomy hemorrhage (B/C), n (%)  **Grade C** | 42 (8.6)  21 (4.3) |
| Bile leakage (B/C), n (%)  **Grade C** | 38 (7.9)  11 (2.3) |
| Chyle leakage (B/C), n (%) | 8 (1.6) |
| Incisional Surgical Site infection, n (%)  Superficial  Deep incisional | 15 (3.1)  14 (2.9) |
| **Length of initial hospital stay**, days, median [IQR] | 12 [8-20] |
| **Readmission within 30 days, n (%)** | 72 (14.8) |
| **90-day reoperation, n (%)** | 70 (14.4) |
| in-hospital/30-day mortality, n (%) | 13 (2.7) |
| 90-day mortality, n (%) | 17 (3.5) |
| *Oncological outcome for PDAC* |  |
| R0 resection, n (%) | 82 (47.1) |
| Lymph nodes total, median [IQR] | 20 [14-26] |
| Lymph nodes involved, median [IQR] | 2 [1.0-5.8] |
| Maximum tumor size, mm, median [IQR] | 27 [21-35] |

**Table legend**: RPD = robotic pancreatoduodenectomy; n = number of patients; % = percentage; IQR = interquartile range; PDAC = pancreatic ductal adenocarcinoma; mm = millimeters.
